# Cranial arterial patterns of the alpaca (Camelidae: *Vicugna pacos*)

**DOI:** 10.1098/rsos.160967

**Published:** 2017-03-22

**Authors:** Haley D. O'Brien

**Affiliations:** Department of Anatomy and Cell Biology, Oklahoma State University Center for Health Sciences, 1111 West 17th Street, Tulsa, OK 74107, USA

**Keywords:** artiodactyla, carotid rete, rete mirabile caroticum, vasculature

## Abstract

Artiodactyl cranial arterial patterns deviate significantly from the standard mammalian pattern, most notably in the possession of a structure called the carotid rete (CR)—a subdural arterial meshwork that is housed within the cavernous venous sinus, replacing the internal carotid artery (ICA). This relationship between the CR and the cavernous sinus facilitates a suite of unique physiologies, including selective brain cooling. The CR has been studied in a number of artiodactyls; however, to my knowledge, only a single study to date documents a subset of the cranial arteries of New World camelids (llamas, alpacas, vicugñas and guanacoes). This study is the first complete description of the cranial arteries of a New World camelid species, the alpaca (*Vicugna pacos*), and the first description of near-parturition cranial arterial morphology within New World camelids. This study finds that the carotid arterial system is conserved between developmental stages in the alpaca, and differs significantly from the pattern emphasized in other long-necked ruminant artiodactyls in that a patent, homologous ICA persists through the animal's life.

## 1. Background

Living camelids are divided into two subfamilies within the Camelidae: the Camelini, or Old World camels (e.g. bactrian and dromedary camels), and the Lamini, or New World camels (llamas, alpacas, guanacoes and vicuñas). Although it is known that camelids possess a carotid rete (CR) [1–9], there are few complete cranial arterial descriptions for this clade, outside of dromedaries. In particular, there are no current, widely available descriptions of the entire cranial arterial system of New World camelids, in spite of behaviours, habitats and growing economic uses that lend import to thorough and accurate documentation of camelid cranial arteries. Formerly distributed throughout a variety of environments, including temperate regions of North America and Europe, living members of the Camelidae are now found in specialized habitats. The geographical ranges of Lamini camelids are restricted to the Andes Mountains of South America, where they are high altitude specialists and routinely encounter extremes in temperature and lengthy periods without meteoric water [10–13]. This oxygen and water depravation, as well as the extremely high and low environmental temperatures these animals routinely encounter can result in negative consequences for unspecialized mammals; as such, New World camelids have a number of physiological specializations for coping with the hypoxia and low atmospheric pressures endemic to high altitudes, particularly within the cardiopulmonary system [14–17]. The CR, a cranial arterial meshwork well developed in artiodactyls, may facilitate some of these extreme aspects of Lamini ecology.

The CR is a subdural arterial meshwork that supplies the majority of oxygenated blood bound for the brain of nearly all artiodactyls. The rete is housed within the cavernous venous sinus, which receives venous blood that has been warmed or evaporatively cooled by the maxilloturbinates [18–21]. The high surface area of the arterial meshwork enables rapid heat exchange between the arterial and pooled venous blood. The result of this exchange is thermal conditioning of the blood bound for the brain, which in turn leads to a suite of physiological consequences that may benefit high-altitude artiodactyl species. First, the CR may protect the brain from altitude hypoxia, as suggested by Caputa [21]. Second, in the arid habitats of Camelini, the CR plays a role in selective brain cooling [22,23]. Continuity between the carotid and ophthalmic retia [4] may further play a role in moderating the temperature of the globe of the eye to protect the animal's vision [24,25]. Overall, living camelids probably benefit from the increased water economy furnished by CR-mediated hypothalamic cooling [26–35]. In spite of the range of plausible physiological benefits imparted by possession of a CR, there is currently, to my knowledge, no comprehensive documentation of the cranial arteries of the Lamini outside of a detailed description of the intracranial cerebral arterial circle by Kiełtyka-Kurc *et al*. [9].

Additionally, camelids are notable for their elongated necks––morphology that may result in atypical haemodynamic and developmental patterns. Although doubt has been cast on a haemodynamic role for the CR in long-necked artiodactyls [36], ontogenetic changes have been documented in the cranial arterial patterns of other long-necked artiodactyls, such as giraffes [37]. Fully mature adult artiodactyls frequently lack an internal carotid artery (ICA) even though arterial development begins from the same embryonic scaffolding as other mammals [38–40]. With the exception of tragulids and perhaps dromedaries [4,9,41,42], the artiodactyl ICA diminishes in diameter or obliterates at a variable point during ontogeny. Some documented artiodactyls lose a patent internal carotid in prenatal ontogenetic stages (*Sus scrofa scrofa* and *Giraffa camelopardalis* [37,39,43–46]), others maintain a patent vessel until shortly after parturition (*Capra hircus hircus*, *Ovis aries* [46]) and still others possess a patent internal carotid until sexual maturity (*Bos taurus*, *Bubalis bubalis* [46–48]). Evidence within Old World camelids (*Camelus dromedarius*) suggests that the ICA may persist through the animal's entire life [1,2,4,6,9,39]. Although the developmental mechanism driving the elimination of the artiodactyl ICA is currently unknown, emergent patterns from the few documented taxa suggest that patency is maintained further into life in artiodactyls with larger body sizes or longer gestation periods, with the exception of giraffes [37]. This study aims to fully describe adult and neonatal cranial arterial patterns of the alpaca, *Vicugna pacos*, in order to document structures important for oxygen metabolism and thermoregulation, as well as to establish a baseline for potential ontogenetic shifts in arterial morphology within a long-necked and large-bodied artiodactyl.

## 2. Material and methods

Four cadaveric alpaca heads were obtained from the collection of S. Williams at Ohio University: two mature males, one mature female and one near-term stillborn of unknown sex. All specimens died of natural causes during the course of unrelated research studies. No animals were sacrificed for the purpose of this study. Shortly after death, the adult alpacas were stored frozen and the stillborn alpaca was preserved in formaldehyde. Data collection follows the methods of Holliday *et al.* [49] and O'Brien & Williams [50], wherein specimens are injected with a radiopaque injection medium, computerized tomography (CT) scanned and then digitally rendered in three dimensions. These more recently derived methods allow soft and hard tissue interactions to be examined in tandem, without the need to destroy either material. For all specimens, either the right or left common carotid artery (CCA) was cannulated (18-gauge angiographic cannula for the stillborn specimen or ⅛-inch PVC tubing (Nalgene) in the adult specimens). Cannulae were fixed in place with surgical ligature and adhesive. The arterial system was then manually flushed with warm water for 10 min, followed by perfusion with 90–300 ml of 10% One-Point anticoagulant solution (depending on specimen size). A higher concentration of anticoagulant was used to flush the formalin-preserved specimen to assist removal and breakdown of blood clots (33% One-Point). Following initial specimen preparation, radiopaque latex vascular injection was conducted, following the criteria for complete perfusion outlined in O'Brien & Williams [50]. All specimens were manually injected with a solution of 40% Liquid Polibar Plus barium sulfate suspension (BaSO4, E-Z-Em, Westbury, NY) in 60% red liquid latex injection medium (Ward's, Rochester, NY). Perfusion continued until latex emerged from the contralateral CCA. The volume of injected medium ranged from 5 ml in the stillborn specimen to 30 ml in the adult specimens. Acetic acid (10% glacial acetic acid solution) was used to set any extravasated latex. For clarity in digital rendering, the venous system was not perfused.

Following radiopaque latex injection, specimens were CT scanned at the Holzer Clinic in Athens, Ohio, on a Philips Brilliance 64-slice CT scanner. Scan resolution for all specimens was 0.67 mm slice thickness, 150 kV and 80 mA, yielding an initial voxel size of 0.693359 × 0.693359 × 0.5. The resultant data were up-sampled to improve resolution in Avizo (version 7.0; VSG). Grey-scale values were averaged across voxels and smaller specimens were up-sampled to a size of 0.1 × 0.1 × 0.1 mm, whereas larger specimens were up-sampled to 0.3 × 0.3 × 0.3 mm. Up-sampling decreases the average voxel size without affecting the inherent quality of the data. This technique generates a visually smoother surface upon reconstruction. Because a 40% barium solution yields stark contrast between hard tissues (cartilage and bone), the skull and arteries were segmented based on distinctive grey-scale values. Manual segmentation was then employed to verify the accuracy of the model. Segmented morphology was then rendered in three dimensions with minimal use of smoothing algorithms (setting of 2 on a scale of 10). Note that vascular nomenclature largely follows that codified in the Nomina Anatomica Veterinaria (2012), with accepted terminology in Latin following the first reference to a vessel.

## 3. Description: results and discussion

### 3.1. Cranial arteries of the adult alpaca

#### 3.1.1. Branches of the external carotid artery

**Figure 1.**
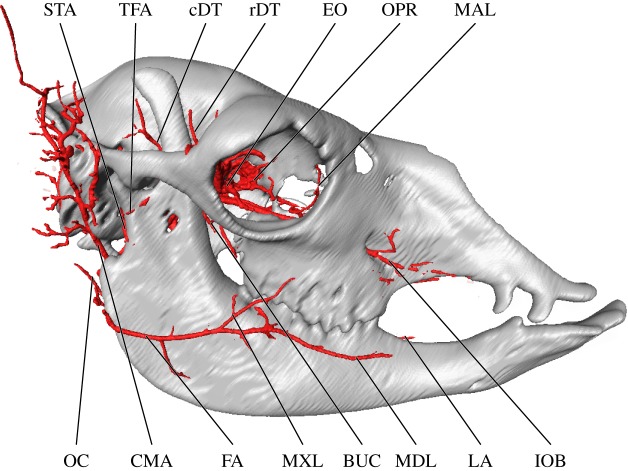
Superficial and orbital arteries of the head of the alpaca, *Vicugna pacos*, lateral perspective. BUC, buccal artery; cDT, caudal deep temporal artery; CMA, ‘common’ auricular artery; EO, external ophthalmic artery; FA, facial artery; IOB, infraorbital artery; LA, lingual artery; MAL, malar artery; MDL, mandibular labial artery; MXL, maxillary labial artery; OC, occipital artery; OPR, ophthalmic rete; rDT, rostral deep temporal artery; STA, superficial temporal artery; TFA, transverse facial artery.

**Figure 2.**
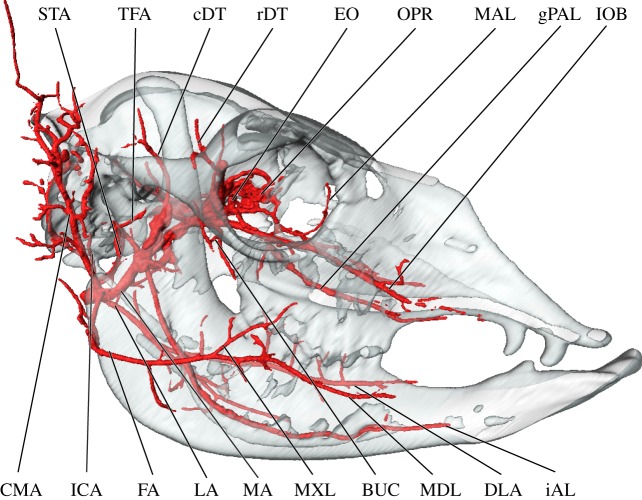
Superficial and orbital arteries of the head of the alpaca, *Vicugna pacos*, lateral perspective. Skull transparent to expose deeper arteries. Note the ICA extending from the carotid artery to the braincase and the enlarged ophthalmic rete (OPR). BUC, buccal artery; cDT, caudal deep temporal artery; CMA, ‘common’ auricular artery; DLA, deep lingual artery; EO, external ophthalmic artery; FA, facial artery; gPAL, greater palatine artery; iAL, inferior alveolar artery; ICA, internal carotid artery; IOB, infraorbital artery; LA, lingual artery; MA, maxillary artery, MAL, malar artery; MDL, mandibular labial artery; MXL, maxillary labial artery; OPR, ophthalmic rete; rDT, rostral deep temporal artery; STA, superficial temporal artery.

**Figure 3.**
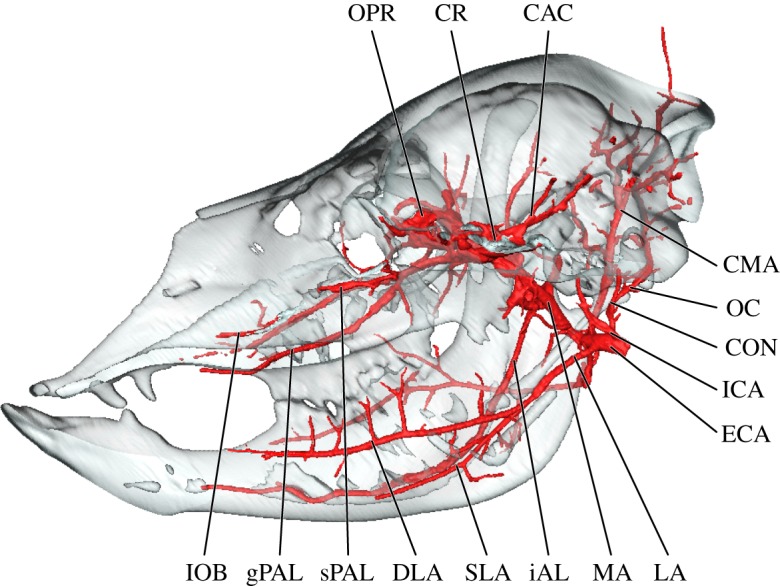
Sagittal section of the skull and arteries of the alpaca, *Vicugna pacos*. CAC, cerebral arterial circle; CMA, ‘common’ auricular artery; CON, condylar artery; CR, carotid rete; DLA, deep lingual artery; ECA, external carotid artery; gPAL, greater palatine artery; iAL, inferior alveolar artery; ICA, internal carotid artery; IOB, infraorbital artery; LA, lingual artery; MA, maxillary artery; OC, occipital artery; OPR, ophthalmic rete; SLA, sublingual artery; sPAL, sphenopalatine artery.

**Figure 4.**
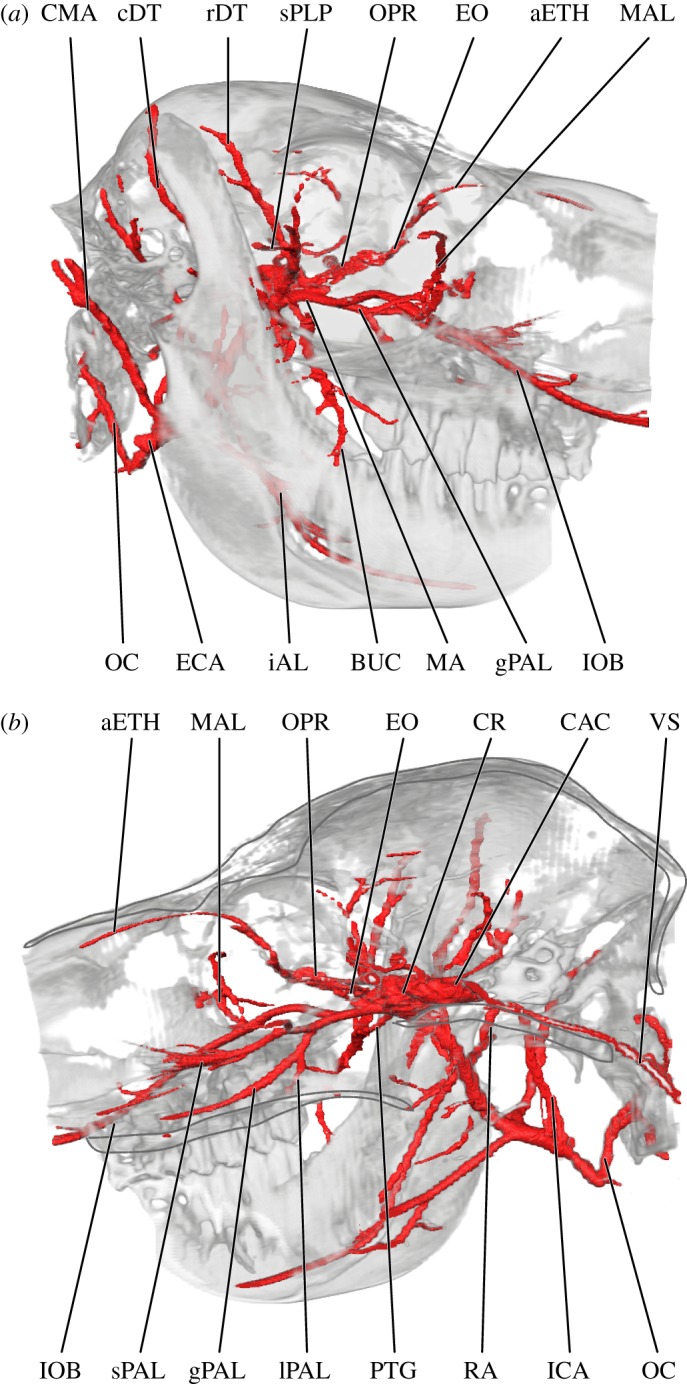
Orbital and palatine arteries of the alpaca, *Vicugna pacos*, from lateral (*a*) and (*b*) medial views. aETH, anterior ethmoidal artery; BUC, buccal artery; CAC cerebral arterial circle; cDT, caudal deep temporal artery; CMA, ‘common’ auricular artery; CR, carotid rete; ECA, external carotid artery; EO, external ophthalmic artery; gPAL, greater palatine artery; iAL inferior alveolar artery; ICA, internal carotid artery; IOB, infraorbital artery; lPAL, lesser palatine artery; MA, maxillary artery; MAL, malar artery; OC, occipital artery; OPR, ophthalmic rete; PTG, pterygoid branches; RA, ramus anastomoticus; rDT, rostral deep temporal artery; sPAL, sphenopalatine artery; sPLP, superior palpebral artery; VS, ventral spinal artery.

Overall, the branching patterns of cranial arteries were conserved among adult specimens. The branches of the external carotid artery (ECA) of the alpaca are summarized in the electronic supplementary material, table S1 and visualized in figures 1–4. The ECA (*arteria [a.] carotis externa*) begins to branch extensively deep to the condylar process of the mandible. The first major branch of the ECA is the occipital artery (*a. occipitalis*; figure 1). From the superior surface of the ECA, the occipital artery shares a short trunk with the ICA (*a. carotis interna*) and the condylar artery (figures 1–3). The trunk uniting these arteries is variable—the vessels may arise in close proximity to each other or from a common trunk, as described (figures 1–3). As the occipital artery ascends, it scours the deep surface of the jugular process and the posterior surface of the temporal crest (mastoid contribution; *crista supramastoidea*). The artery terminates by splitting into smaller branches that permeate the occipital region (nuchal ligament (*ligamentum nuchae*) and muscles) and a larger caudal meningeal artery (figures 1 and 2). The latter enters the cranium via a large mastoid foramen (*foramen mastoideum*) before radiating across the caudal half of the meninges. The ICA itself is reduced in calibre, and the vessel does not leave a medial bullar groove as it ascends to the basicranium as in other artiodactyls with a homologous ICA (figures 2 and 3; see e.g. *Moschiola* [42]; *Tragulus* [51]). As the ICA enters the basicranium, it traverses the promontorial foramen, coursing within a carotid canal in close association with the promontorium of the petrosal. The ventral surface of the petrosal is scoured by a slight transpromontorial sulcus. This sulcus corresponds to direct contact by the ICA of non-artiodactylan mammals [52–57]; however, in the adult alpaca, the ICA does not make direct contact with the petrosal. The presence of a transpromontorial sulcus in adult alpacas may be an effect of arterial reduction during ontogeny (see below). Upon entering the cranium, the ICA leaves a notch at the rostral-most extent of the epitympanic wing (*sensu* [55,57]; this structure is also referred to as the ‘pole of the promontorium’ *sensu* [58] and the ‘anteromedial flange’ *sensu* [59]). Once inside the brain case, the ICA communicates with the CR (figures 2–4; also illustrated for other Lamini by Kiełtyka-Kurc *et al*. [9]).

Following the occipital and ICA branches, a large artery arises from the superolateral surface of the ECA, immediately posterior to the tympanohyal (*os tympanohyoideum*). This artery is highly dendritic and supplies a large region equivalent to the distribution of the caudal auricular artery of other artiodactyls (*a. auricularis caudalis*), as well as some of the typical distribution of the superficial temporal (*a. temporalis superficialis*) and caudal masseteric arteries (*a. masseterica profunda*; figure 2). The majority of branches from this parent artery supply the auricular region, without ramification by additional, individual auricular arteries (figure 2). As such, a more accurate term for this vessel may be the ‘common auricular artery’. After branching from the ECA, the common auricular artery then ascends between the jugular process and the posterior surface of the auditory bulla, scoring the superior surface of the mastoid, caudal to the external acoustic meatus. Ventral to the external acoustic meatus, the common auricular artery distributes a posterolateral zygomatic branch that ramifies the temporomandibular joint (TMJ) and contributes extensive, smaller arteries to the posterosuperior border of the masseter, a region typically supplied by caudal branches from the superficial temporal artery [60]. A stylomastoid artery (*a. stylomastoidea*) departs from the medial surface of the common auricular artery at the level of the stylomastoid foramen. Finally, at the level of the crista supramastoidea, the common auricular artery splits into rostral and caudal terminal branches. This rostral branch is not homologous with the *a. auricularis rostralis* proper, which derives from the superficial temporal artery in non-camelid artiodactyls [46,60]. The caudal termination of the common auricular artery supplies the posterior scalp/superior occipital region as well as the cartilaginous pinna of the ear. The rostral division of the common auricular artery supplies the remainder of the ear and gives off a proper branch to the posterior portion of the temporalis muscle.

In the alpaca, the lingual and facial arteries do not form a common linguofacial trunk (*truncus linguofacialis*), as the facial artery (*a. facialis*) has a separate origin on the lateral wall of the ECA (figure 2). The facial artery has a somewhat deviant course relative to other artiodactyls and many other mammals, in that it approaches the superficial facial region from the caudal border of the mandibular ramus (*ramus mandibulae*) instead of the ventral border of the mandible (*corpus mandibulae margo ventralis*). In this respect, its course is somewhat reminiscent of the transverse facial artery (*a. transversa facei*). The facial artery transmits a number of small vessels to the digastric, masseter and buccinator muscles, providing a substantial volume of the blood supply to the muscles supporting the oral cavity. It terminates by splitting into superior and inferior labial arteries (*a. labialis superior* and *a. labialis inferior*).

The lingual artery (*a. lingualis*) is the third and anterior-most major branch of the ECA, departing from the ventral surface of the parent vessel caudal to the greater horn of the hyoid (figures 2–4). It distributes through the parenchyma of the tongue as expected, but has an aberrant branching pattern to ramify regions of the ventral mandibular border typically supplied by branches of the facial artery. Near the emergence of the lingual artery from the ECA, the sublingual artery (*a. sublingualis*) parts from the lingual artery (figures 2 and 3). The sublingual artery of the alpaca is relatively large and compensates for the absence of the submental artery. In addition to supplying the sublingual gland, the sublingual artery courses along the ventral border of the mandible, supplying the floor of the mouth and the lingual vestibule. The final branch of the ECA is a greatly reduced and partially perfused superficial temporal artery (figures 1 and 2). The typical distribution of the superficial temporal artery is ramified by a sizeable branch of the ‘common auricular artery’ (described above; figures 1 and 2). The transverse facial artery originates on the anterior aspect of the superficial temporal artery and courses obliquely to curve around the caudal border of the mandibular ramus near the TMJ (*articulation temporomandibularis*; figures 1 and 2). It perfuses a small quadrant of the masseter and sends a terminal ramus to the capsule of the TMJ.

#### 3.1.2. Branches and distribution of the maxillary artery

The branches and distribution of the alpaca maxillary artery (MA) are summarized in the electronic supplementary material, table S2 and visualized in figures 1–4. Common to contemporary artiodactyls, the MA (*a. maxillaris*) is the major source of oxygenated blood to the brain (figures 3 and 4; [47]). The only source of collateral flow to the cerebral arterial circle (*circulus arteriosus cerebri*) is through the ventral spinal artery (figure 4). The MA distributes blood to the brain (via the CR), pterygoid muscles, palate, nasal cavity, oral cavity, ethmoidal region, frontal region, cranial sinuses, superficial facial structures above the maxillary tuberosity (including the upper lip and fleshy portion of the rostrum), the maxillary dentition, the dentary (including mandibular alveoli), and the lower lip and chin (figures 1–4). The caudal deep temporal (cDT) artery (*a. temporalis profunda caudalis*) is the first major branch to arise as a dorsal branch from the MA (figures 1, 2 and 4). This arterial offshoot courses deep to the posterior border of the coronoid process of the mandible, ultimately perfusing the temporalis muscle. The cDT artery shares a common origin with the inferior alveolar and masseteric arteries (*a. alveolaris inferior*; *a. masseterica*), which proceed inferior and lateral to the MA, respectively (figures 1, 2 and 4). The inferior alveolar artery enters the mandibular canal via the mandibular foramen. As it courses through the inferior alveolar canal, the alveoli of the mandibular dentition are supplied before the artery ultimately exits the mental foramen. The superficial distribution of the artery is to the lower lip and chin. The masseteric artery separates from the MA/cDT complex dorsal to the inferior alveolar artery. After separating from the MA, the masseteric artery hooks around the neck of the condylar process, after which it supplies the lateral facial/zygomatic region and its eponymous muscle (figures 1 and 2). The dorsal termination supplies the TMJ (figure 2). Deep to the coronoid process, the buccal artery (*a. buccalis*) emerges from the lateral surface of the MA (figures 1, 2 and 4). This small artery traces the ventral surface of the zygomatic process before bifurcating into the inferior palpebral artery (*a. palpebralis inferior*) and a branch to the buccinator muscle between the coronoid process and the posterior border of the maxilla.

Distal to the cDT artery on the dorsal surface of the MA, a variable number of rami connect the MA to the CR through the foramen orbitorotundum. It is through these *arteria anastomotica* that the brain receives almost all of its oxygenated blood (figures 2–4). The alpaca CR is further ramified via the MA by a *ramus anastomoticus* that, as in true ruminant artiodactyls (Pecora), enters the brain case through the foramen ovale, as well as by a reduced but patent ICA as described above (*ramus anastomoticus*: figure 4; ICA: figures 2–4). Additional collateral contributions to the CR of the alpaca include the vertebral arteries (figure 4; *a. vertebralis*; via the ventral spinal artery (*a. spinalis ventralis*)— the vertebral arteries do not make a direct contribution to intracranial circulation). The cerebral arterial circle and blood supply to the brain of camelids is discussed in detail by Kiełtyka-Kurc *et al*. [9], and will therefore not be duplicated here. These authors identify variation within cerebral arterial circle vessels for both New and Old World camelids [9]. The MA then anastomoses freely with the ophthalmic rete (OPR; figures 2–4; *rete mirabile ophthalmicum*). Near the pterygoid crest, the rostral deep temporal artery (*a. temporalis profunda rostralis*) departs the MA from within this periorbital anastomotic network. Immediately caudal to the OPR, the rostral deep temporal artery proceeds superiorly along the anterior temporal line, sending perforating branches into the anterior border of the temporalis muscle (figures 1, 2 and 4). Also within this region, several pterygoid branches (*rami pterygoidei*) depart the internal surface of the MA (figure 4). These branches have a short course before perfusing the pterygoid muscles. The rostral termination of the MA is tripartite: the external ophthalmic (EO) artery departs medially (*a. opthalmica interna*) and the malar artery superiorly (*a. malaris*), with the infraorbital artery (*a. infraorbitalis*) continuing anteriorly (figures 2–4). The infraorbital artery has two divisions: a lateral division, which continues through the infraorbital canal, and a medial division (the descending palatine artery; *a. palatina descendens*), which serves as the parent vessel for the greater, lesser and sphenopalatine (*a. palatina major, a. palatina minor* and *a. sphenopalatina*, respectively) arteries, which supply the soft and hard palates, the nasal septum, internal nasal vestibule and turbinates (figure 4).

#### 3.1.3. Arterial blood supply to the eye and orbit

The arterial blood supply to the eye and orbit of the alpaca are summarized in the electronic supplementary material, table S3 and imaged predominantly in figure 4. The CR extends rostrally, outside of the braincase, to participate in the formation of an OPR through which the eye and periorbita are supplied (figure 4). The eyeball is directly supplied by the central artery of the retina (*a. centralis retinae*), which originates as a derivative of the cerebral arterial circle and exits the optic foramen within the optic nerve. The remainder of the globe of the eye is supplied by the internal ophthalmic artery (*a. ophthalmica interna*), which originates from an anastomosis between the cerebral arterial circle and the CR. The EO artery (*a. ophthalmica externa*) condenses from an extensive network of rami among the MA and ophthalmic and carotid retia (figures 1–4). From this meshwork, the EO artery consolidates prior to transecting the orbital region (figure 4). It supplies the periorbita, extraocular muscles and ethmoidal region (figure 4). The ciliary trunk and ciliary vessels (*aa. ciliares*) are not perfused. Direct branches of the MA supply the superficial periorbita. The lacrimal artery (*a. lacrimalis*) is a small division of the MA, which arises slightly proximal to the carotid/OPR complex. It courses laterally towards the closed post-orbital bar, where it divides into lacrimal and superior palpebral branches (*a. palpebralis superior*). Collateral circulation to the superficial orbit is by the malar artery, which originates from the infraorbital artery (figures 1, 2 and 4). Prior to entering the orbital portion of the infraorbital canal, the infraorbital artery gives off the tortuous malar artery. The malar follows the anterior margin of the orbit, contacting the lacrimal bone, and exiting the orbit via a notch near the lacrimal fossa. In addition to the superficial orbit, the malar artery perfuses the caudal portion of the face.

The infraorbital artery is the rostral continuation of the MA. The MA bifurcates into palatine and infraorbital arteries near the common orbital tendinous ring and courses ventral to the periorbita. The orbital portion of the infraorbital artery has few branches, but gives rise to the malar artery, as described above. The infraorbital artery then proceeds to the rostrolateral facial region through its eponymous canal. Upon exiting the infraorbital foramen, the infraorbital artery branches extensively across the lateral nasal and superior labial regions.

### 3.2. Cranial arterial patterns of the stillborn alpaca

**Figure 5.**
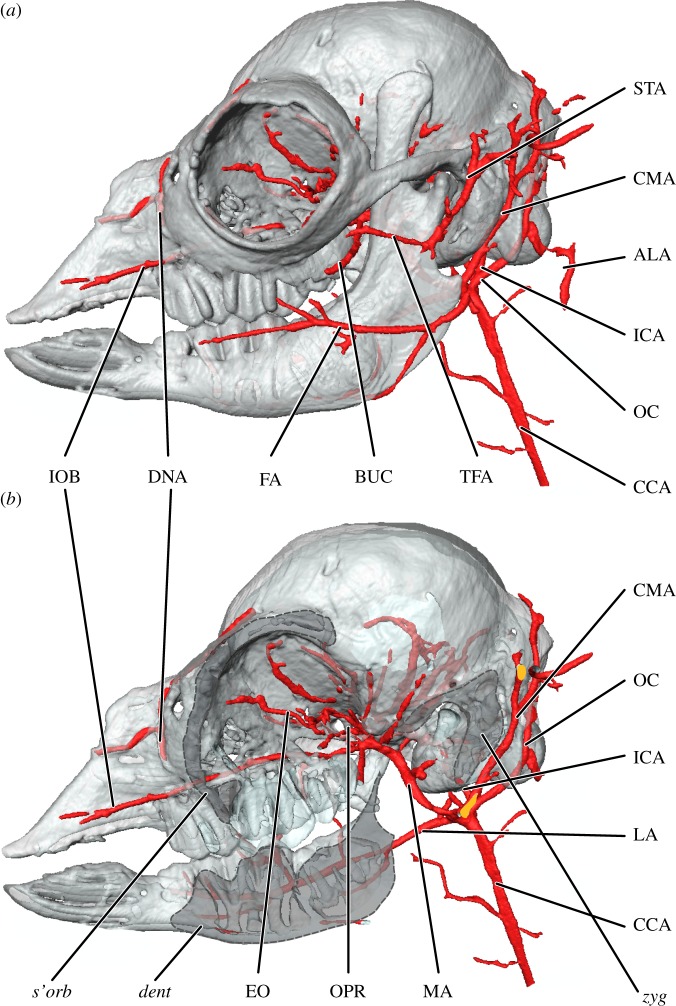
Cranial arteries of the stillborn alpaca, *Vicugna pacos*, (*a*) superficial lateral perspective and (*b*) deep lateral perspective with supraorbital (*s'orb*), dentary (*dent*) and zygomatic (*zyg*) bones cut. Sectioned arteries are indicated in orange. Perfusion did not extend rostrally beyond the skull, into the soft tissue structures of the nasal and labial regions. ALA, alar artery; BUC, buccal artery; CCA, common carotid artery; CMA, ‘common’ auricular artery; DNA, dorsal nasal artery; EO, external ophthalmic artery; FA, facial artery; ICA, internal carotid artery; IOB, infraorbital artery; LA, lingual artery; MA, maxillary artery; OC, occipital artery; OPR, ophthalmic rete; STA, superficial temporal artery; TFA, transverse facial artery.

A single stillborn alpaca was available as a representative of early developmental stages. The stillborn alpaca (age estimated as proximate to full gestation) had been preserved in formalin for several years prior to injection. As such, the arterial walls were hardened and several distributing arteries (infraorbital, labial and dorsal nasal) did not perfuse into the rostral-most soft tissues (figure 5). The stillborn alpaca specimen included both head and neck, providing an opportunity to describe the branches of the CCA (*a. carotis communis*) low in the neck (figure 5).

#### 3.2.1. Arteries of the neck

The arteries of the neck of the stillborn alpaca are summarized in the electronic supplementary material, table S4, and all arteries are presented in figure 5. Deviations in CCA patterning across ontogeny may indicate an arterial mode of postural hypertension mitigation endemic to long-necked artiodactyls, such as giraffes [37]. Of particular interest is an anastomosis between enlarged occipital and vertebral vessels that courses through the alar foramen of the atlas—the ‘alar artery’, first noted in giraffes [61] (*a. alaris*, *sensu* [37]). In a description of the cerebral arteries of the closely related Old world camel, *Camelus dromedarius*, Kanan [4] notes the presence of such an anastomosis, and, indeed, an alar artery is present branching from the CCA of the stillborn *Vicugna*. This vessel therefore follows the same course in all three long-necked artiodactyl species (*Camelus* [4]; *Giraffa* [37]; *Vicugna*, figure 5*a*).

Unlike the condition described for developing giraffes (stillborn and 6 month old; [37]), the stillborn alpaca retains a large, fully patent ICA (figure 5*a*,*b*). The ICA arises near the occipital artery from a common point on the superior surface of the CCA, thus providing a precise demarcation between the CCA and the ECA. The ICA of the stillborn alpaca makes direct contact with the petrosal, unlike the adult in which the carotid canal is incompletely filled by the ICA. Below this transition, the cranial thyroid (*a. thyroidea cranialis*) and descending pharyngeal (*a. pharyngea descendens*) arteries arise from the anterior surface of the CCA (in order from proximal to distal). The descending pharyngeal artery originates close to the hyoid, superior to the larynx.

#### 3.2.2. Branches of the external carotid artery and course of the internal carotid artery

The branches of the ECA of the stillborn alpaca are imaged in figure 5. The ECA is the rostral continuation of the CCA after the occipital, condylar and internal carotid arteries depart the dorsal surface of the parent artery near the jugular process. In the stillborn alpaca, the ECA primarily supplies the superficial face and scalp, and the deeper regions of the cranium below the level of the zygomatic bone (e.g. floor of the oral cavity, portions of the cranial base). The ICA is a substantial and easily identifiable branch of the CCA that arises as the caudal-most branch from a common origin with the occipital and condylar arteries. The ICA briefly courses anteriorly, medial to the occipital and condylar vessels, before ascending towards the tympanic floor on the deep surface of the large tympanic bulla. This association leaves a groove on the medial wall of the bulla—a groove that is not present in fully developed adult skulls. As the ICA courses in close proximity to the petrosal, the artery makes direct contact with the bone, leaving a groove that remains in the adult even as the artery becomes relatively reduced in calibre. Within the open, developing tympanic floor, the ICA courses antero-superiorly before anastomosing with the posterior extent of the CR on the internal surface of the basisphenoid, definitively anterior to the petrosal.

The remaining arteries follow the same pattern between the stillborn and adult specimens, and the branching patterns described in the electronic supplementary material, tables S1 through to S3 are therefore not replicated. Interestingly, the ophthalmic and carotid retia are already reasonably well developed in the stillborn specimen. The full formation of the OPR in early ontogeny may be owing to the fact that the ophthalmic vessels condense from large vascular plexuses consisting of the primordial ophthalmic, nasal, ethmoidal and cerebral vessels [62]. The extent of the CR may result from a different mechanism, which requires further investigation on comparative anatomical and proximate, mechanistic scales. Compared to other non-camelid artiodactyls, the CR of the stillborn alpaca, although well developed, is somewhat smaller than expected at parturition. This may be owing to the presence of a patent ICA. By birth, *Sus*, *Ovis, Capra* and *Giraffa* possess retia that occupy nearly the entire floor of the basicranium, in the absence of an ICA [37,39,46,63]. Contrarily, in *Bos* and *Bison*, which maintain patent ICA's at birth [46,48], the CR does not yet fill the compartment lateral to the sella turcica––a similar extent to that of the stillborn alpaca. Wible [39] notes that early artiodactyl embryos maintain a tubular ICA associated with the intracranial surface of the basisphenoid. These studies suggest that the ICA is initially formed in artiodactyls, but degenerates or becomes stenotic across development. This degeneration may result in hypoxia to the lateral sellar compartment. Hypoxia is a known mechanism for the recruitment of vascular endothelial growth factors [64]. The potential linkage between ICA obliteration, hypoxia and rete formation should be explored in further detail and in a mechanistic context to resolve whether rete ontogeny is influenced by intrinsic developmental processes, or extrinsic factors, such as altitude and environment.

## 4. Conclusion

Digital anatomical data collection and dissection techniques indicate the presence of a patent, functional ICA in both stillborn and mature alpacas. This conclusion supports recent work performed by Kiełtyka-Kurc *et al*. [9], who describe a well-formed ICA present across both New World and Old World camelids. The functional presence of this vessel is unusual in mature artiodactyls, as suid and pecoran artiodactyls are known to obliterate this vessel throughout ontogeny [1,2,4,37,39,43–48]. Overall, the major cranial arterial branching patterns of the alpaca do not differ significantly from those studied in a comparative context by Kiełtyka-Kurc *et al*. [9], nor do they differ significantly from the patterns detailed for the domestic dromedary [4,6,65]. New and Old World camelids inhabit strikingly different habitats which may promote their own unique demands on the animals' cardiovascular systems. That there are few major discrepancies between the arteries supplying the head and CR of these animals suggests that the cranial vascular patterns of camelids reflect phylogeny rather than function. Because camelids are frequently reconstructed as the earliest group of extant artiodactyls to originate [66–69], the maintenance of a patent ICA in this group suggests a possible stepwise loss of this artery throughout Artiodactyla. This phenomenon should be validated on a clade-wide scale.

The stillborn specimen described in this study provides additional data regarding the timing of artiodactyl cranial artery patterning. In the alpaca, the adult cranial arterial pattern is established prior to birth, contrary to the gradual, post-natal obliteration of the ICA in *Bos* and *Bubalis* [46,48]. A similar phenomenon of an ontogenetically conserved pattern in the arteries supplying the brain has also been noted recently for giraffes; however, for giraffes, the ICA is absent from an early age [37], rather than persistent throughout life as in camelids. These developmental patterns are strikingly different, in spite of the fact that giraffids and camelids have long gestational periods (greater than 325 days; [70,71]) and face a similar cardiovascular burden owing to neck elongation. A paucity of data on anatomical variation, developmental timing and genetic signalling necessitates further studies to assess whether these patterns arise through macroevolutionary, physiological and/or environmental mechanisms. Untangling the pattern and process underlying the evolution and development of the CR may help elucidate how this group came to occupy highly variable habitats—including the extreme environments preferred by alpacas and other camelids. Low specimen numbers should be interpreted conservatively, and future studies should focus on the inclusion of additional specimens, particularly earlier ontogenetic stages.

## Supplementary Material

Table S1. Branches and distribution of the external carotid artery of the adult alpaca, Vicugna pacos

## Supplementary Material

Table S2. Branches and distribution of the maxillary artery of the adult alpaca, Vicugna pacos

## Supplementary Material

Table S3. Blood supply to the eye and orbit of the adult alpaca, Vicugna pacos

## Supplementary Material

Table S4. Arteries in the neck of the stillborn alpaca, Vicugna pacos. Model 1. Adult Alpaca Arteries: https://figshare.com/s/014c954981edf40377b7 Model 2. Adult Alpaca Skull: https://figshare.com/s/5df961807eb70cf345d0 Model 3. Stillborn Alpaca Arteries: https://figshare.com/s/bb133750f346d5cb01e9 Model 4. Stillborn Alpaca Skull: https://figshare.com/s/c76b2958f99fc7687fff

## Data accessibility

3D.stl files are available at Figshare. Files can be opened with Preview for Mac or with a number of freeware programs and sites (e.g. http://www.viewstl.com/). *Adult Alpaca Arteries:* http://dx.doi.org/10.6084/m9.figshare.3185095 *Adult Alpaca Skull:* http://dx.doi.org/10.6084/m9.figshare.3185086 *Stillborn Alpaca Arteries:* http://dx.doi.org/10.6084/m9.figshare.3185074 *Stillborn Alpaca Skull:* http://dx.doi.org/10.6084/m9.figshare.3185068.
