# Supplementary material for: Cranial arterial patterns of the alpaca (Camelidae: Vicugna pacos)
Source: R Soc Open Sci. 2017 Mar 22;4(3):160967. doi: 10.1098/rsos.160967 (PMC5383842; doi:10.1098/rsos.160967)
Supplement: Table S1. Branches and distribution of the external carotid artery of the adult alpaca, Vicugna pacos [file rsos160967supp1.pdf]

**Table S1: Tributaries of the External Carotid Artery of the Alpaca, *Vicugna Pacos***			
**Artery**	**Origin**	**Course**	**Distribution**
External Carotid	Common Carotid	Begins at bifurcation with ICA; courses throughout deep and superficial structures of the head.	Superficial structures of the face; lingual and pharyngeal structures of the deeper cranium
Descending Pharyngeal	External Carotid	Branches from ECA near caudal border of mandible; courses ventrally to oropharynx.	Oropharynx
Internal Carotid	External Carotid	Branches from superior surface of the ECA in close proximity to the occipital artery. Ascends toward basicranium without branching and enters braincase via carotid foramen and canal. Anastomoses with caudal portion of carotid rete.	Carotid rete
Occipital	External Carotid	From superior surface of ECA, gives off condylar a. shortly after departing ECA; as it ascends, scours deep surface of the jugular process and posterior surface of temporal crest/mastoid bone. Terminates in by bifurcating into 1) smaller branches that perfuse the occipital region (Nuchal muscles and ligament); 2) caudal meningeal (enters cranium via mastoid foramen).	Occipital region; terminates in occipital parenchyma and dura mater intracranially as caudal meningeal
Common Auricular	External Carotid	Branches from superolateral aspect of ECA just behind the tympanohyal; ascends between jugular process and posterior surface of tympanic bulla; scores surface of mastoid posterior to tympanic bulla/external acoustic meatus (EAM). Ventral to the EAM, splits into posterolateral zygomatic branch (also ramifies temporomandibular joint); stylomastoid artery branches from medial surface of parent vessel at level of stylomastoid foramen; at level of the crista supramastoidea, splits into rostral and caudal auricular arteries.	Posterior scalp/superior occipital region; rostral auricular branch also supplies posterior temporalis m.
Lingual	External Carotid	Third and anteriormost major branch of the ECA; departs caudal to the greater horn of the hyoid; courses anteriorly through parenchyma of tongue	Parenchyma of tongue, lingual glands

Facial	External Carotid	Branches from inf. aspect of common auricular artery, courses obliquely to curve around the posterior border of the mandible just ventral to the angular process; Gives off small branches to digastric and masseter muscles; hooks around mandible and courses anteriorly along the mandibular corpus; throughout anterior course supplies lateral muscles of mastication	Supplies digastric m.; masseter and buccinator; deep branches to buccinator; terminates by splitting into superior and inferior labial arteries
Superficial Temporal	External Carotid	Short vessel with reduced distribution. Branches from ECA near the caudal border of the mandible. Contributes superficial temporal artery and rami to the temporomandibular joint.	Lateral aspect of the face near zygomatic, temporalis m., and temporomandibular joint
Transverse Facial	Superficial Temporal	Small, anterior-coursing branch of the superficial temporal. Supplies posterosuperior quadrant of the masseter muscle and the temporomandibular joint capsule.	Masseter m. and temporomandibular joint capsule
Condylar	Occipital	Medial branch off occipital; enters cranium at condylar foramen.	n/a; incompletely perfused
Caudal Meningeal	Occipital	After entering the cranium through the enlarged mastoid foramen, the caudal meningeal divides into a meningeal branch and a petrous portion of the temporal canal branch.	Caudal meninges; petrosal branch ramifies middle ear (stylomastoid artery)
Stylomastoid	Common Auricular	Branches from medial surface of common auricular; enters stylomastoid foramen; courses through facial canal.	Middle ear
Mandibular Labial	Facial	Facial bifurcates at mandibular angle into maxillary and mandibular labial	Caudal portion of upper lip; rostal portion supplied by infraorbital artery
Maxillary Labial	Facial	Facial bifurcates at mandibular angle into maxillary and mandibular labial	Lower lip
Lingual	External Carotid	Third and anterior-most major branch of the ECA; departs caudal to the greater horn of the hyoid; courses anteriorly through parenchyma of tongue	Parenchyma of tongue, lingual glands

Sublingual	Deep Lingual	Departs deep lingual laterally, shortly after the bifurcation of the parent artery from the lingual; follows interal border of mandible to sublingual gland target	Sublingual gland
Deep Lingual	Lingual	Terminal bifurcation of lingual artery; follows division of intrinsic tongue musculature and mylohyoid (extralingual) musculature	Floor of oral cavity, inferior to tongue
