# Supplementary material for: Cranial arterial patterns of the alpaca (Camelidae: Vicugna pacos)
Source: R Soc Open Sci. 2017 Mar 22;4(3):160967. doi: 10.1098/rsos.160967 (PMC5383842; doi:10.1098/rsos.160967)
Supplement: Table S2. Branches and distribution of the maxillary artery of the adult alpaca, Vicugna pacos [file rsos160967supp2.pdf]

Table S2: Branches and Distribution of the Maxillary Artery of the Alpaca, *Vicugna pacos*			
**Artery**	**Origin**	**Course**	**Distribution**
Maxillary Artery	External Carotid Artery	Anterior continuation of the ECA; Nomenclature shifts following the departure of the superficial temporal artery/common auricular artery from the dorsal surface of the ECA. Three segments, including proximal, orbital, and distal.	Majority of cranial arterial distribution; Supplies brain, meninges, pterygoid, palate, nasal cavity, oral cavit, ethmoidal region, frontal region, cranial sinuses, the face above the maxillary tuberosity, the dentary, and the lower lip and chin
Inferior Alveolar	Maxillary	Ventral branch of maxillary a.; enters mandibular canal via mandibular foramen; courses through canal, supplying alveoli of mandibular dentition; exits mental foramen	Alveoli of mandibular dentition; chin
Masseteric	Maxillary	Dorsal to the inferior alveolar a., masseteric separates from the MA and perfuses the masseter after hooking around the neck of the condylar process; extensive branching within lateral facial region; dorsal termination supplies TMJ	masseter; lateral zygomatic region; TMJ
Caudal Deep Temporal	Maxillary	From superior surface of the maxillary a., deep to the coronoid process; caudal deep temporal follows posterior border of coronoid superiorly	posterior border of the temporalis
Rostral Deep Temporal	Maxillary/CR/Oph. Rete	Departs maxillary a. near pterygoid crest; immediately caudal to ophthalmic rete, artery proceeds superiorly, following the anterior temporal line	anterior border of temporalis m.
Ramus anastomoticus	Maxillary	Extensive branching in region of foramen orbitorotundum	Carotid and orbital retia

Carotid Rete	Maxillary	Occupies lateral sellar compartment of cranium; forms smooth floor of braincase / internal surface of the basisphenoid	Cerebrum and cerebellum
Orbital Rete	Maxillary	Proximal portion of external opththalmic	Eyeball and some periorbita
Pterygoid Branches	Maxillary	Internal surface MA, short course before reaching pterygoid bone; branches extensively into pterygoid muscles; small branch enters foramen on posterior surface pterygoid process	pterygoid muscle and bone
Buccal	Maxillary	small, lateral branch of IMA, departs deep to coronoid process; courses toward malar; ventral to zyg. pr of maxillary bone, splits into ventral and superior branches; part of superior = inferior palpebral; ventral branch toward buccinator	pterygoid, maxillary, and buccinator muscles; lower eyelid
(Caudal) Lateral Nasal	Buccal	From buccal artery, courses under orbital process and traverses crista facialis	Lateral nasal region
External Ophthalmic	Maxillary/Opthalmic Rete	Rami from maxillary a. to carotid and ophthalmic rete = extensive, largely indistinguishable; Ext. ophthalmic forms extensive rete, crosses and supplies periorbita, extraocular muscles, and ethmoidal region	Periorbita and ethmoidal region
Lacrimal	Maxillary	Small lateral division of internal maxillary; slightly proximal to carotid + ophthalmic rete; courses laterally toward post-orbital bar, where divides into lacrimal and superior palpebral branches	lacrimal gland, superior eyelid

Infra-Orbital	Maxillary	Lateral division of the anterior terminal branches of maxillary; MA bifurcates into palatine and IOA near common orbital tendinous ring; courses ventral to periorbita with few branches; dorsal nasal departs prior to entry of parent artery into IO canal; courses through IO canal, exits facial IO foramen and branches extensively in lateral nasal/superior labial region	Lateral facial region
Descending Palatine	Greater Palatine	Departs G. Pal between malar and pterygoid; descends into caudal palatal region	Caudal hard palate, soft palate
Greater Palatine	Maxillary	Part of the anterior terminal bifurcation of the internal maxillary artery; IMA is shorter in alpacas than for other mammals; division occurs near common tendinous orbital ring; exits orbital region through pterygopalatine foramen (medial foramen on the maxilla); courses through hard palate and enters oral cavity through greater palatine foramen	
Nasopalatine	Greater Palatine	Superomedial division of Greater palatine; courses along the floor of the nasal cavity and nasal septum toward the incisive fossa	
