# Supplementary material for: Cranial arterial patterns of the alpaca (Camelidae: Vicugna pacos)
Source: R Soc Open Sci. 2017 Mar 22;4(3):160967. doi: 10.1098/rsos.160967 (PMC5383842; doi:10.1098/rsos.160967)
Supplement: Table S3. Blood supply to the eye and orbit of the adult alpaca, Vicugna pacos [file rsos160967supp3.pdf]

Table S3: Arterial Blood Supply to the Eye and Orbit of the Alpaca, *Vicugna pacos*			
**Artery**	**Origin**	**Course**	**Distribution**
External Ophthalmic	Maxillary Artery / Opthalmic Rete	Rami from maxillary a. to carotid and ophthalmic rete = extensive, largely indistinguishable; Ext. ophthalmic forms extensive rete, crosses and supplies periorbita, extraocular muscles, and ethmoidal region	Periorbita and ethmoidal region
Internal Ophthalmic	Carotid rete and cerebral arterial circle	Anastomosis between CAC and carotid rete; Through the optic foramen and around the optic nerve	Eyeball and periorbita
Lacrimal	Maxillary	Small lateral division of internal maxillary; slightly proximal to carotid + ophthalmic rete; courses laterally toward post-orbital bar, where divides into lacrimal and superior palpebral branches	Lacrimal gland, superior eyelid
Inferior palpebral	Buccal	From termination of buccal artery, courses superiorly, around inferior postorbital bar, toward lower eyelid	Lower eyelid
Superior Palpebral	Lacrimal	See above	Superior eyelid
Dorsal Nasal	Infraorbital	Prior to entering the orbital portion of the infraorbital canal, parent artery gives off tortuous dorsal nasal a.; dorsal nasal follows anterior margin of orbit, contacting lacrimal bone, leaves orbit via notch near lacrimal fossa	Caudal, dorsal nasal region
Infra-Orbital	Maxillary	Lateral division of the anterior terminal branches of maxillary; MA bifurcates into palatine and IOA near common orbital tendinous ring; courses ventral to periorbita with few branches; dorsal nasal departs prior to entry of parent artery into IO canal; courses through IO canal, exits facial IO foramen and branches extensively in lateral nasal/superior labial region	Lateral facial region
