# Supplementary material for: Cranial arterial patterns of the alpaca (Camelidae: Vicugna pacos)
Source: R Soc Open Sci. 2017 Mar 22;4(3):160967. doi: 10.1098/rsos.160967 (PMC5383842; doi:10.1098/rsos.160967)
Supplement: Table S4. Arteries in the neck of the stillborn alpaca, Vicugna pacos. Model 1. Adult Alpaca Arteries: https://figshare.com/s/014c954981edf40377b7 Model 2. Adult Alpaca Skull: https://figshare.com/s/5df961807eb70cf345d0 Model 3. Stillborn Alpaca Arteries: https://figshare.com/s/bb133750f346d5cb01e9  [file rsos160967supp4.pdf]

Table S4: Arteries in the Neck of the Stillborn Alpaca, *Vicugna pacos*			
**Artery**	**Origin**	**Course**	**Distribution**
Common Carotid		Bears same carotid-vertebral-occipital anastomosis as is present in giraffes at level of alar foramen of atlas.	Continues as external carotid artery; directly supplies structures in the neck
External Carotid	Common Carotid Artery	Begins at trifurcation of occipital, condylar, and internal carotid arteries.	Face, tongue, auricle, occiput, superficial temporal region
Internal Carotid	External Carotid Artery	Begins from common trunk with occipital and condylar arteries; Ascends toward anterior lacerate foramen. Courses anteriorly through carotid canal, just rostral to petrosal. Directly supplies posterior extent of developing carotid rete.	Carotid rete; cerebral arterial circle. Rete anastomoses anteriorly with rami from maxillary artery
Cranial Thyroid	Common Carotid Artery	From anterior surface of Common Carotid Artery, descends toward thyroid gland.	Thyroid Gland
Descending Pharyngeal	Common Carotid Artery	Small, poorly perfused stalks from medial wall of Common Carotid Artery may contribute descending pharyngeal; Proximal to occipital artery.	Pharynx
Caudal Auricular	External Carotid	As in adult; all arterial flow to auricle appears to be through "Common Auricular" artery.	Auricle
